# Skin organoids as a new biological standard

**DOI:** 10.1093/burnst/tkag033

**Published:** 2026-04-24

**Authors:** Imaan Ahmed, Abbas Shafiee

**Affiliations:** Faculty of Health, Medicine and Behavioural Sciences, Frazer Institute, The University of Queensland, Brisbane, QLD 4102, Australia; Faculty of Health, Medicine and Behavioural Sciences, Frazer Institute, The University of Queensland, Brisbane, QLD 4102, Australia; Metro North Hospital and Health Service, Queensland Health, Brisbane, QLD, 4029 Australia

Early *in vitro* models of human skin included two-dimensional cell cultures using keratinocytes and fibroblasts. Though cost-effective and simple to produce, these monolayers fail to recapitulate the skin’s three-dimensional structure. Further progress saw the layering of keratinocytes on top of fibroblasts, to create a three-dimensional ‘skin equivalent’. These could be increased in anatomical and functional relevance with the addition of other populations, such as dermal papilla cells to induce hair growth. However, hair growth in these *in vitro* constructs was often incomplete. Furthermore, these constructs lacked the diverse cell populations present within the human skin organ (Supplementary Table 1).

It was therefore a remarkable breakthrough in the field when the human skin organoid (SKO) was developed by Lee *et al*. [1]. The SKO, generated from human induced pluripotent stem cells (hiPSCs), was created via modulating pathways that drive skin development in human embryogenesis. The resulting organoid self-organized into a cyst-like skin structure, with the epidermis forming on the inner layer of the cyst and the dermis forming on the outer layer, complete with hair follicles, melanocytes, and neural populations (Figure 1). This SKO was assessed to be equivalent of craniofacial human skin within the second trimester and represented a significant milestone in the *in vitro* modelling of skin.

Since then, SKOs have been utilized for various applications. Fascinatingly, hiPSC-derived SKOs were shown to promote healing in a mouse model of frostbite, when combined with a gelatine-hydrogel [2]. Another exciting application of SKOs for regenerative medicine is transplantation. The hiPSC source allows SKOs to be generated from patient cells, creating an autologous, personalized therapeutic.

One study generated SKOs from patients with recessive dystrophic epidermolysis bullosa, then used the SKOs to isolate both keratinocytes and fibroblasts for creating *in vitro* skin constructs [3]. This method not only generates patient-specific models that can be used for preclinical testing, but it also provides a way to upscale and bank patient-derived cells for transplantation, overcoming the availability issues and limited proliferative capacity of primary cells. Similarly, SKOs have been used as a source from which skin-derived precursors (SKPs) could be isolated for potential hair regenerative therapeutics [4]. Cells with characteristic SKP spheroid morphology were isolated from SKOs and expressed markers indicative of hair-inductive capacity.

SKOs also make valuable models of infectious disease. One study developed a model of atopic dermatitis by co-culturing SKOs with *Staphylococcus aureus* [5]. SKOs infected with *S. aureus* developed a disrupted epidermal skin barrier, a key feature of atopic dermatitis, and increased in inflammatory cytokine production. SKOs have also been applied for modelling viral infection, by using the Mpox virus [6].

The SKO is, however, not without limitations. The original SKO differentiation protocols lacked several aspects of skin architecture, namely vasculature, immune populations, and sweat glands, making modelling of inflammatory and immunological conditions challenging. Studies have aimed to address these through optimizations of the SKO protocol. SKOs with simple, tubular gland-like structures in the dermis that possessed a marker expression profile characteristic of eccrine sweat glands were generated [7, 8]. The morphology and quantity of the hair follicles could also be enhanced through culture on an air–liquid interface [9]. Additionally, by incorporating hiPSC-derived vascular organoids with resident immune populations into the SKO, a vascularized SKO was established (Figure 1) [10].

Although these SKOs overcome key limitations of the original model, their efficacy as disease models or for drug screening has yet to be investigated. Furthermore, as SKOs resemble foetal skin, they do not yet possess features characteristic of adult skin, such as terminal hair follicles and apocrine sweat glands. Nevertheless, the vascularized SKO is a model with great potential. As the entire vascularized SKO can be generated from a single hiPSC source, this method has the potential to allow patient-specific and highly physiologically relevant skin to be developed *in vitro* for personalized medicine. Additionally, as the vascularized SKO possesses immune populations, the modelling of inflammatory and autoimmune conditions becomes more achievable.

Another difficulty is achieving consistency between SKOs, both within and between experiments. Heterogeneity has often been noted in SKO morphology, such as with off-target cartilage formation and the distribution of hair follicles [1, 5, 8]. This may be in part due to the methodology of manually culturing SKOs in welled plates, which results in slight differences in growth factor concentration due to pipetting error and differing well evaporation rates (edge effect). Even these slight concentration differences may have an impact on the developing SKO [5]. The use of a bioreactor or an automated liquid handling system for culturing SKOs should be investigated, as it may reduce variation and subsequently improve SKO reproducibility.

Other sources of variability between SKOs arise from differences among hiPSCs lines. Different hiPSCs show intrinsic variability in gene regulatory states, epigenetic memory, and signalling pathway activity, which subsequently influences their responsiveness to exogenous growth factor modulation [5]. If generating SKOs from a particular pluripotent stem cell line is unsuccessful, it may require optimizations to the differentiation process. For example, sequential optimization for the differentiation factors allows the SKO differentiation process to be tailored to the specific hiPSC line [5]. Other efforts to reduce SKOs variability include modulating the Wnt signalling pathway to inhibit off-target cartilage differentiation, as well as shortening the differentiation process to minimize the impact of differing pluripotent stem cell line proliferation rates [5]. Adjusting the hiPSC expansion conditions using growth factors and matrix culture systems can also improve organoid competence in the weaker cell lines. In addition, early aggregate size strongly affects oxygenation, diffusion, patterning, and lineage balance, suggesting that each cell line may need its own aggregate size optimization [7, 8].

With SKOs becoming an increasingly popular model for *in vitro* skin, the question of nomenclature for SKOs becomes relevant. Prior to the introduction of the hiPSC-derived SKO, the term ‘human SKO’ was used to describe other constructs, such as bilayer models composed of keratinocytes and fibroblasts. Though these are useful models and were the available approximations for SKOs previously, we suggest that the term ‘SKO’ be clarified. Organoids are defined as three-dimensional (3D) models that assemble by self-organization and mimic the architecture and function of the respective organ. The hiPSC-derived SKOs developed by Lee *et al*. in 2020, and the subsequent refinements of this model [1, 4, 5, 7, 8], therefore more accurately represent a true ‘human SKO’ than previous constructs, as they recapitulate important features such as hair follicles and neural networks.

The term SKO is currently used inconsistently across the literature and often includes a wide range of self-organized skin-like structures, engineered skin equivalents, and multicellular aggregates. To improve clarity and facilitate comparison across studies, we therefore propose a minimal set of criteria for defining a model as a ‘skin organoid’. The bona fide SKO should meet three core features: (i) development through intrinsic self-organization of stem/progenitor cells rather than purely scaffold-guided assembly; (ii) multilineage differentiation encompassing the principal epithelial and mesenchymal skin compartments, and (iii) a three-dimensional architecture recapitulating the spatial organization of native skin including a stratified epidermal layer supported by dermal tissue. More advanced integrated SKOs may incorporate additional levels of physiological complexity, including the formation of skin appendages (e.g. hair follicles or sweat glands), as well as vascular, or immune components that recapitulate skin microenvironmental interactions characteristic of the skin ecosystem.

Human SKOs are a recent breakthrough, with substantial opportunity for further development. A potential application is to combine SKOs with bioengineering techniques. Microfluidic culture systems may allow SKOs to grow larger with increased hair production and epidermal differentiation. Further application of bioengineering principles may similarly improve SKO morphology and longevity, by facilitating nutrient diffusion and better recapitulating the extracellular environment using biomimetic extracellular matrices and dynamically controlled microphysiological systems. Dynamic culture systems, such as bioreactors, improve nutrient delivery and reduce uneven distribution, hypoxia, and handling related variability, thereby minimizing stochastic difference in organoid growth and maturation.

**Figure 1:**
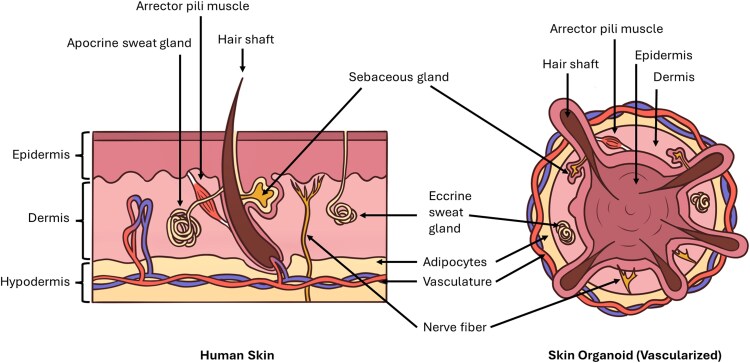
A schematic comparing bona fide human skin to vascularized skin organoids.

Additionally, directed patterning and microengineering technologies may enable the transition towards planar, spatially organized tissue architecture with compartmentally defined epidermal, dermal, and appendageal domains. Achieving this level of structural fidelity would close the gap between self-organizing SKOs and fully engineered human skin equivalents.

Another field where SKOs could be applied is cosmetic testing. The use of animals for testing cosmetics is challenging due to inherent differences with human skin and ethical concerns. Furthermore, as the United States Food and Drug Administration phases out mandatory animal testing, human *in vitro* models become increasingly relevant. Physiological properties of the SKO, such as skin barrier integrity, sensitization reactions, elasticity, and appendage function, require investigation and standardization. For application in cosmetic and safety testing, SKO platforms must demonstrate reproducibility across laboratories, standardized manufacturing protocols, well-defined performance metrics, and predictive validity compared with the available *in vitro* or clinical benchmarks. Therefore, SKO-based assays must be compatible with high-throughput and quality-controlled workflows and demonstrate relevance to human skin physiology and toxicological responses.

Limitations, such as the inverse cystic morphology, which currently requires manual manipulation to correct, and the fragile nature of the organoid skin, make the application of topical agents like creams difficult. Furthermore, the foetal nature of the SKO needs to be taken into consideration when testing products for use on adult skin. Methods need to be designed to further mature the SKO *in vitro*, such as via organotypic slice culture to improve media exposure and reduce necrosis, as has been done in other organoids. Nevertheless, the SKO represents a scalable, physiologically relevant human model that could reduce the demand for animal testing in the future.

Finally, SKOs also hold significant potential for modelling non-infectious skin disease. As has already been achieved for recessive dystrophic epidermolysis bullosa, the hiPSC source allows SKOs to be derived from patients with genetic skin conditions [3]. This application could facilitate *in vitro* modelling of other conditions such as heritable ichthyosis, or conditions with both environmental and genetic components, such as psoriasis. Not only would the SKO provide a platform to elucidate the mechanisms driving these conditions, but it would also provide a disease model to test therapeutics.

SKOs are shifting skin research from fragmented, single-component models to integrated systems that more faithfully capture the complexity of human skin. As outlined above, SKOs are rapidly emerging as powerful platforms for disease modelling, drug discovery, therapeutic testing, and regenerative medicine. Achieving the SKOs full potential requires overcoming current limitations in maturation, reproducibility, and standardization. Advances in bioengineering, gene editing, and the integration of immune and vascular components will further expand the capabilities of SKOs in areas ranging from cosmetic safety testing and precision dermatology, to modelling of rare genetic skin diseases. Together, SKOs represent a powerful experimental platform for advancing the understanding of human skin biology and the rapid development of next-generation therapies.

## Supplementary Material

Supplementary_Materials_table_1_tkag033
