# Supplementary material for: Skin organoids as a new biological standard
Source: Burns Trauma. 2026 Apr 24;14:tkag033. doi: 10.1093/burnst/tkag033 (PMC13251885; doi:10.1093/burnst/tkag033)
Supplement: Supplementary_Materials_table_1_tkag033 [file supplementary_materials_table_1_tkag033.docx]

**Skin Organoids as a New Biological Standard**

***Supplementary Table 1****: Human skin organoids vs. reconstructed skin models*

| **Feature** | **Skin organoids** | **Reconstructed skin models** |
| --- | --- | --- |
| **Origin** | Derived from hiPSCs or ESCs | Primary cells from donors |
| **Cellular complexity** | High. Including multiple cell lineages | Low to moderate. Usually, keratinocytes and fibroblasts |
| **Structural organization** | 3D self-organizing structures mimicking embryonic/fetal skin development | Bioengineered layer(s) |
| **Presence of skin appendages (e.g., hair follicles, glands)** | May contain early-stage appendages | Absent |
| **Physiological relevance** | High. Mimics fetal skin development | Low to moderate. Resembles adult skin function |
| **Immune competence** | Emerging; immune cells can be integrated | Absent or limited |
| **Vascularization** | Emerging; vascularized models in development | Emerging; vascularized models in development |
| **Level of biological integration** | Integrated, self-organizing multicellular systems | Component-based models lacking higher-order tissue integration |
| **Scalability for drug screening** | Moderate. Not yet high-throughput ready | High. Widely used in industry |
| **Time to generate** | Weeks to months | Days to weeks |
| **Customization / disease modeling** | High. Can model patient-specific genetic diseases | Low. Difficult to model specific genetic conditions |
| **Reproducibility** | Variable. Sensitive to culture conditions | High. Standardized protocols exist |
| **Regulatory acceptance** | Still under development | Established and accepted in testing guidelines (OECD, etc.) |
| **Applications** | Developmental biology, disease modeling, regenerative medicine | Toxicology testing, cosmetics, drug permeability studies |
| **Key strengths** | Physiologically relevant, customizable, suitable for mechanistic studies | Reproducible, fast, validated for regulatory use |
| **Key limitations** | Limited maturity, complex and time-consuming, batch variability | Lacks complexity, limited cellular diversity and physiological function |
